# Neural feedback strategies to improve grasping coordination in neuromusculoskeletal prostheses

**DOI:** 10.1038/s41598-020-67985-5

**Published:** 2020-07-16

**Authors:** Enzo Mastinu, Leonard F. Engels, Francesco Clemente, Mariama Dione, Paolo Sassu, Oskar Aszmann, Rickard Brånemark, Bo Håkansson, Marco Controzzi, Johan Wessberg, Christian Cipriani, Max Ortiz-Catalan

**Affiliations:** 1 Center for Bionics and Pain Research, Mölndal, Sweden; 2 Department of Electrical Engineering, Chalmers University of Technology, Gothenburg, Sweden; 3 The Biorobotics Institute, Scuola Superiore Sant’Anna, Pisa, Italy; 4 Department of Excellence in Robotics & AI, Scuola Superiore Sant’Anna, Pisa, Italy; 5 Prensilia SRL, Pontedera, Italy; 6 Department of Physiology, Institute of Neuroscience and Physiology, Sahlgrenska Academy, University of Gothenburg, Gothenburg, Sweden; 7 Department of Hand Surgery, Sahlgrenska University Hospital, Gothenburg, Sweden; 8 Clinical Laboratory for Bionic Extremity Reconstruction, Division of Plastic and Reconstructive Surgery, Medical University of Vienna, Vienna, Austria; 9 Department of Orthopaedics, Institute of Clinical Sciences, Sahlgrenska Academy, University of Gothenburg, Gothenburg, Sweden; 10 Center for Extreme Bionics, Biomechatronics Group, MIT Media Lab, Massachusetts Institute of Technology, Cambridge, MA USA; 11 Operational Area 3, Sahlgrenska University Hospital, Mölndal, Sweden

**Keywords:** Motor control, Biomedical engineering

## Abstract

Conventional prosthetic arms suffer from poor controllability and lack of sensory feedback. Owing to the absence of tactile sensory information, prosthetic users must rely on incidental visual and auditory cues. In this study, we investigated the effect of providing tactile perception on motor coordination during routine grasping and grasping under uncertainty. Three transhumeral amputees were implanted with an osseointegrated percutaneous implant system for direct skeletal attachment and bidirectional communication with implanted neuromuscular electrodes. This neuromusculoskeletal prosthesis is a novel concept of artificial limb replacement that allows to extract control signals from electrodes implanted on viable muscle tissue, and to stimulate severed afferent nerve fibers to provide somatosensory feedback. Subjects received tactile feedback using three biologically inspired stimulation paradigms while performing a pick and lift test. The grasped object was instrumented to record grasping and lifting forces and its weight was either constant or unexpectedly changed in between trials. The results were also compared to the no-feedback control condition. Our findings confirm, in line with the neuroscientific literature, that somatosensory feedback is necessary for motor coordination during grasping. Our results also indicate that feedback is more relevant under uncertainty, and its effectiveness can be influenced by the selected neuromodulation paradigm and arguably also the prior experience of the prosthesis user.

## Introduction

Despite significant progress in the field of upper limb prosthetics, robotic devices capable of restoring the dexterity and perception of lost biological arms remain elusive. It has been several decades since researchers started exploring invasive solutions to establish electrical connection with the peripheral nervous system of amputees^1,2^. Invasively interfacing muscles that survived amputation has been shown to provide rudimental but reliable open-loop control of prosthetic hands in activities of daily living^3–5^. On the sensory side, neural interfacing with implanted electrodes has been shown to produce rough but long-term stable somatotopic perception (i.e., sensations felt in the phantom hand)^3,6–8^. Experimental prosthetic systems using implanted electrodes to provide superior control, along with somatosensory feedback, have been typically based on transcutaneous human-machine interfaces. However, issues with safety and reliability of theses interfaces have hindered their translation into clinical practice^9^.

Direct neural stimulation has been proven successful in eliciting tactile-like sensations^2^. Dhillon and Horch showed in 2005 that tactile as well as proprioceptive sensations could be elicited using intraneural electrodes^10^. Since then, other research groups have demonstrated the use of neural stimulation to provide functionally relevant feedback, such as the discrimination of object compliance^11–13^, texture^14^, and finger position^15^, as well as detection of slippage^16^. These studies investigating sensory feedback with implanted electrodes used non-invasive surface electromyography (sEMG) as the signal source for control. sEMG is known to be susceptible to interference, motion artifacts, and environmental factors that can deteriorate the control experience^17^. Therefore, higher performance may be achieved with a more precise and reliable control interface than sEMG. In fact, superior prosthetic function has been reported using implanted electrodes solely for the purpose of control and without sensory feedback ^3–5^. Nevertheless, prosthetic grasping behavior has not been found to approach that of a biological hand, despite reliable open-loop control using implanted electrodes with rich but incidental visual, auditory, and osseoperceptive sensory feedback^18^.

In this study, we investigated if closed-loop control with somatotopically appropriate sensory feedback could restore motor coordination during grasping. Three subjects with transhumeral amputation were implanted with a novel neuromusculoskeletal interface. Direct skeletal attachment of the prosthesis to the body was achieved using a percutaneous osseointegrated implant (OPRA Implant System, Integrum AB, Sweden)^19^. The conventional osseointegration implant system was further engineered to provide bidirectional communication to implanted electrodes in nerves and muscles using a series of feedthrough mechanisms (e-OPRA). Epimysial electrodes were implanted on muscles to be used as source of control, and spiral cuff electrodes placed on the median and ulnar nerves were used to deliver electric stimulation to provide somatosensory feedback.

**Figure 1:**
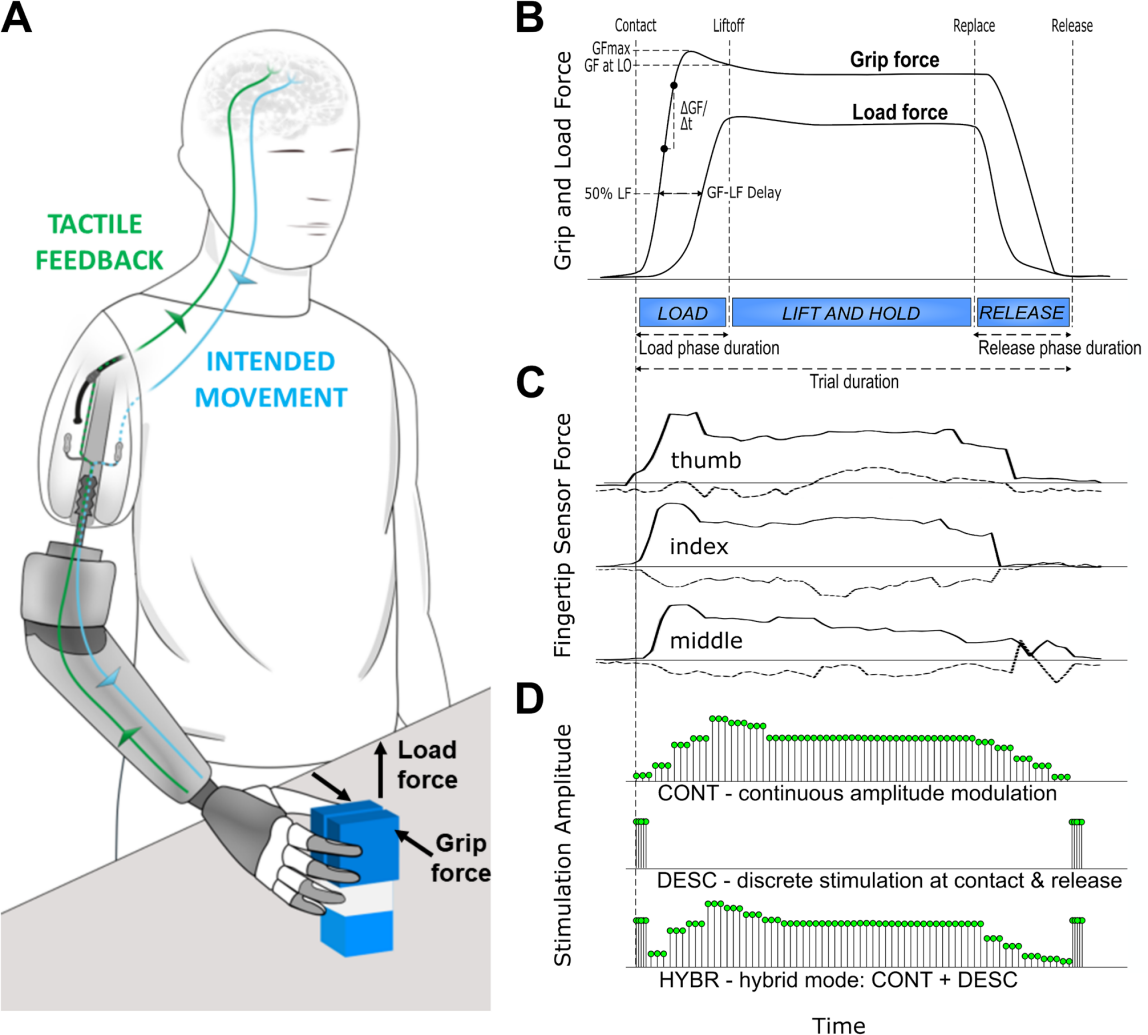
Representation of the closed-loop control and the experiment. (**A)** The subjects performed a repetitive pick and lift test while being provided with somatotopic tactile sensory feedback via extraneural stimulation. **(B)** The instrumented test object measured the grip force (GF) and load force (LF). From this, all the performance metrics were calculated. **(C)** Tactile feedback was based on the normal and tangential forces measured from the sensors in the thumb, index and middle fingers of the robotic hand, not the instrumented object. **(D)** Three different feedback modes were provided in random order: continuous modulation of pulse amplitude (CONT) directly proportional to the forces measured; discrete stimulation with fixed parameters (DESC) corresponding to the discrete events of touch and release; or a hybrid of the two modes (HYBR). Illustration of panel **(A)** by Sara Manca.

Multiple force sensors mounted on a robotic hand drove neural stimulation paradigms that provided the subjects with real-time tactile sensations (Fig. 1). We employed three paradigms of biologically inspired tactile sensory feedback and compared them to the conventional control condition in which subjects received no supplementary but only incidental feedback (no-feedback). These paradigms were inspired by the natural mixture of discrete and continuous afferent signals mediated in the intact human hand by fast and slow adapting mechano-receptors^20^. Discrete mechanical stimulus events, such as touch and release of an object, are typically mediated via bursts of action potentials in afferent fibers, whereas continuous information, such as the grasping force, is mediated in a more continuous fashion. Non-invasive feedback based on a “Discrete Event-driven Sensory Control policy” (DESC) has already been shown to improve prosthesis-grasping in amputees^21^. However, this approach neglects the continuous flow of afferent information that is naturally present while in contact with an object. We wanted to explore the effect of coupling and decoupling these two modes. Considering this, the sensory paradigms employed were: Continuous stimulation with pulse amplitude proportional to grasping and lifting forces; Discrete stimulation corresponding to touch and release events; and a Hybrid between these two (Fig. 1).

The pick and lift test (PLT) with an instrumented object was used to assess motor coordination during (1) routine grasping and (2) under uncertainty. We measured the temporal coordination between grip and load forces applied to the object during the different phases of the picking and lifting task. Uncertainty, which may increase the subjects’ reliance on sensory feedback^22–25^, was introduced by randomly changing the weight of the test object in between grasps^26^. In addition to objective metrics, we assessed the subjective experience arising from the neural stimulation paradigms in terms of quality, intensity, naturalness, and pleasantness of the elicited percepts^27,28^.

## Results

The following results describe the pooled data of all three subjects to point to general trends we observed and to make for easier reading. Wherever necessary or interesting, we further clarify how this applied to or deviates from the individual results. Owing to the small number of subjects, we only made statistical comparisons within subjects, not between. All individual results are available in the text for Experiment 1 and in the Supplementary materials for Experiment 2.

### Sensory characterization

**Figure 2:**
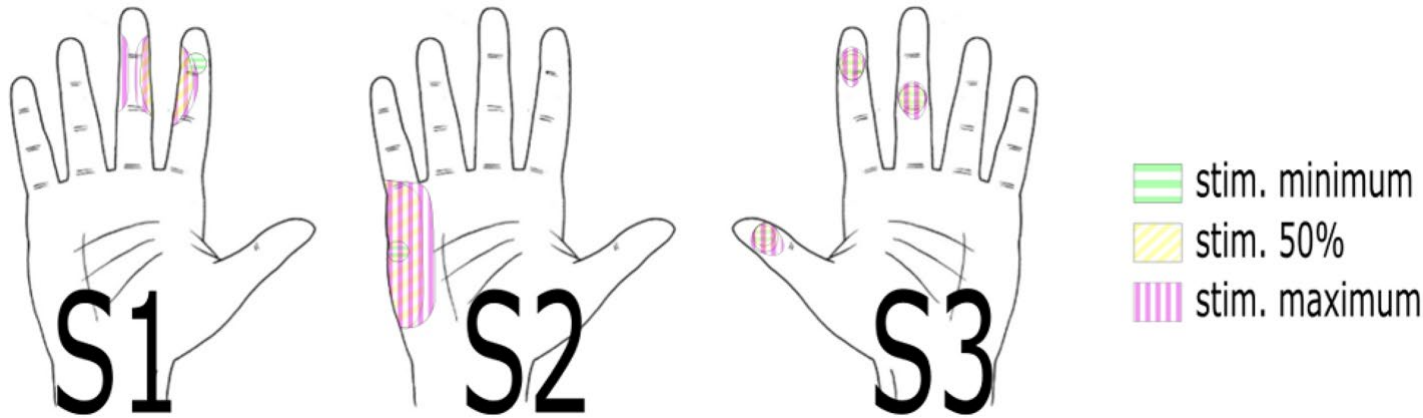
Perception maps drawn by all subjects representing the perceptive areas and locations. The different colors and patterns show the area of perception for the different levels of current amplitude modulation of the stimulation. In green is the area at minimum stimulation (perception threshold), in purple the area at maximum stimulation and in yellow the area at 50% of the stimulation range. S1 and S2 are right-side amputees, S3 is a left-side amputee.

Experiments started with an extensive fitting session aimed to find each subject’s personal stimulation settings to elicit sensory perception. The electrode contact used for stimulation and the stimulation parameters were selected empirically, prioritizing the contact that required the lowest charge to produce perception (Table 1 and Fig. 2). The resulting percepts were located on the middle and distal phalanxes of the index and middle fingers for S1 and S3, and also on the distal phalanx of the thumb for S3. These locations corresponded to the somatotopic arrangement of the median nerve where the selected electrode was located. For S1, and to a lower extent also for S3, an increase in pulse current amplitude was perceived as an increased area of stimulation on the phantom fingers. For S2, whose selected electrode was placed around the ulnar nerve, percepts were located on the palmar side below the fifth finger. The perceived area increased towards the center of the phantom palm when increasing the pulse current amplitude. Higher pulse amplitudes resulted in higher perceived intensities in all subjects. Furthermore, all subjects perceived the phantom hand in the same location as the robotic hand.

**Table 1:** Subjects’ customized parameters for neural stimulation.

	Nerve	Impedance (kΩ)	Pulse width (µs)	Min. pulse amplitude (percept threshold) (µA)	Max. pulse amplitude (modulation ceiling) (µA)	Perceivable amplitude modulation steps
S1	Median	2.5	200	300	650	14
S2	Ulnar	9.4	100	80	140	7
S3	Median	2.6	200	380	550	8

### Experiment 1: motor coordination under certainty

At the beginning of this experiment, subjects were fitted with a custom prosthetic setup. Among other components, this comprised the IH2 Azzurra research robotic hand (Prensilia SRL, Italy), controlled via direct proportional speed control to perform a tridigital grasp.

For each feedback condition, experiments started with a familiarization stage in which the subjects grasped and relocated fragile objects with different weights (200 g, 300 g, 400 g), akin to the virtual eggs test^21^. Subjects then completed two sessions of the PLT (20 repetitions each) to assess motor coordination under certainty, i.e., with a non-breakable object of known and constant weight of 200 g, similar to experiments by Cipriani et al.^29^ who used non-invasive interfaces.

#### Subjects showed high motor coordination with hybrid feedback

**Figure 3:**
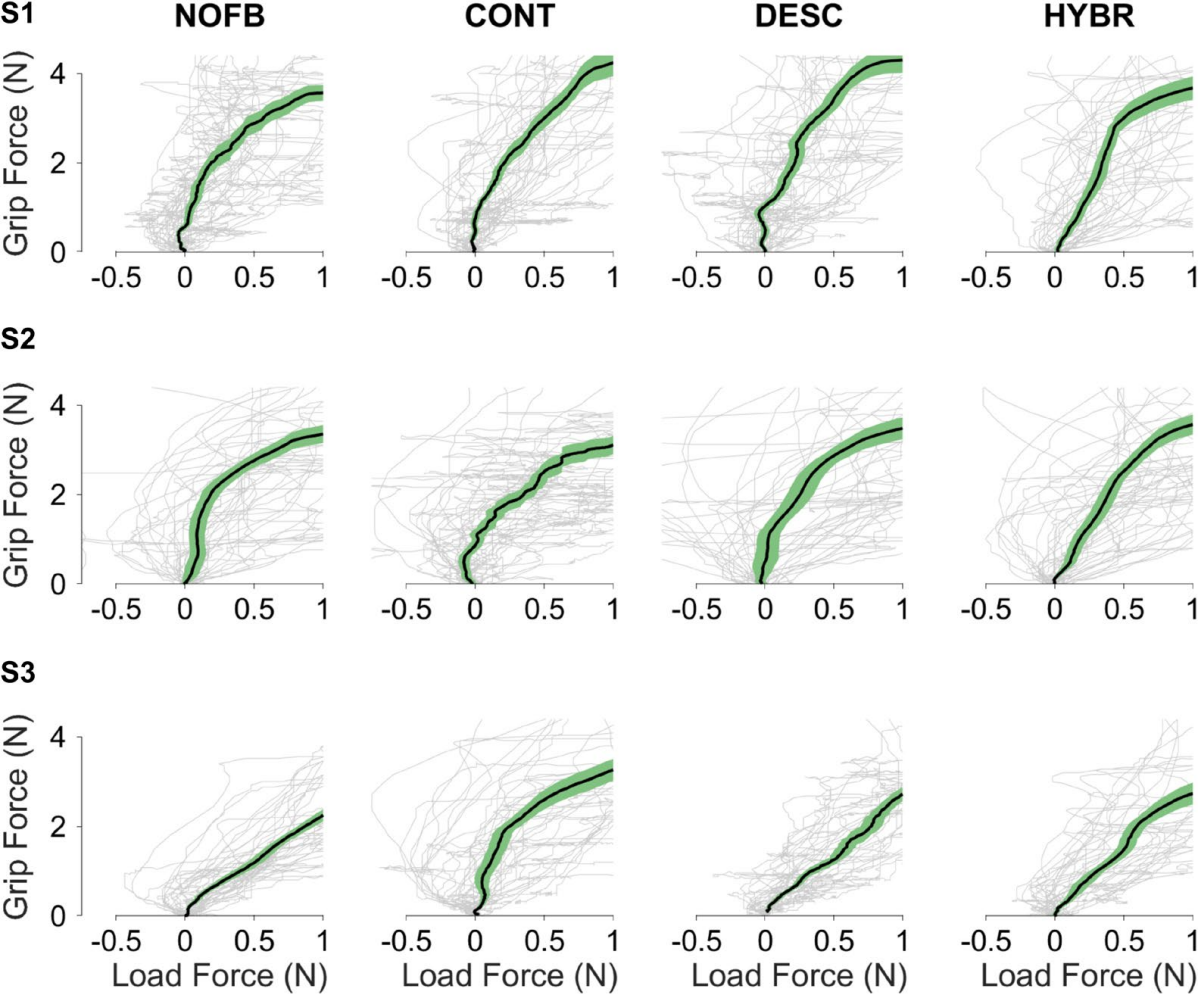
Representation of the motor coordination from Experiment 1. Plots of the grip and load forces for all subjects and all modes. The plot draws the correlation between grip and load forces during the initial part of the grasping and lifting of an object. A more linear relationship between grasp and load forces indicates more natural, mature grasping behavior^31^. The thin gray lines are the individual traces. The thicker line represents the median of all the 40 repetitions and the green areas around it represent the 95% confidence areas.

Motor coordination can be visualized as the temporal correlation of the grip and load forces (Fig. 3). High temporal correlation, that is, a more linear relationship between the temporal evolution of the grasp and lift forces, indicates a more mature and natural grasping behavior^30^. Figure 3 shows qualitatively that all subjects displayed higher motor coordination with *Hybrid* feedback. Notably, S3 generally exhibited high motor coordination even without feedback, which was hindered when provided with *Continuous* feedback.

**Figure 4:**
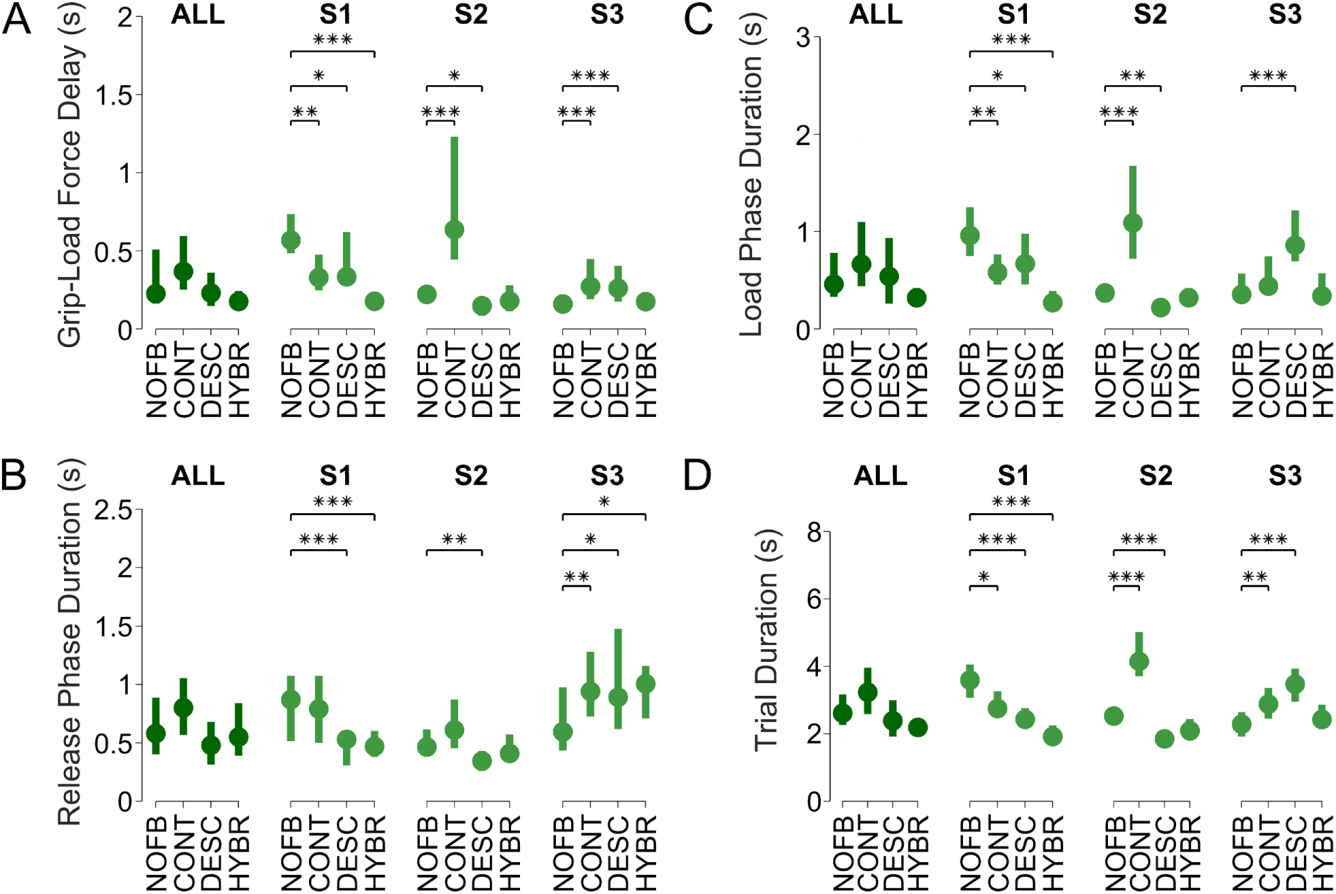
Experiment 1, results from the pick and lift test with same weight. All graphs show boxplots (median and interquartile range) of the pooled data on the left side and the individual results on the right side for all sensory feedback modes. The statistical significance is reported according to the following notation: * = p < 0.05, ** = p < 0.01, *** = p < 0.001. **(A)** Delay between grip and load forces during the load phase. The delay is measured between the instants when grip and load forces reached 50% of the load force measured at lift-off. **(B)** Load phase duration, measured as the time between the first contact and object lift-off. **(C)** Release phase duration, measured as the time between starting to replace the object and the last contact. **(D)** Trial duration measured as the total time between the first and the last contacts.

Motor coordination can also be quantified as the temporal delay between the instants when the grip force and load force reach 50% of the load force at lift-off^21^. Looking at the pooled data, we found that this delay was considerably reduced from 226:347 ms (median:IQR) in the *no-feedback* condition, to 176:122 ms in the *Hybrid* feedback condition, which represents a 22% reduction of the median delay (Fig. 4). However, this trend was mostly represented by, and statistically significant for, S1. Individually, the delay was lowest with *Hybrid* feedback for S1 and second lowest for S2 and S3. The grip-load delay with *Discrete* feedback was 230:215 ms and thus comparable to *no-feedback*, but with *Continuous* feedback it actually increased by 62% (368:344 ms). S2 had the shortest (149:108 ms) and longest (597:802 ms) grip-load forces delays of all subjects, with *Discrete* and *Continuous* feedback, respectively. Remarkably, S3 had the second-shortest result, with a delay of only 160:79 ms, using *no-feedback*.

#### Supplementary feedback did not affect the maximum grip force

No statistically significant difference was found regarding the maximum grip force applied to the test object when using different feedback modes (Figure S1—Supplementary Materials). The peak forces applied were contained within similar ranges (3.9:0.8 N). The grip force measured at the instant of lift-off was 3.6:0.7 N (median:IQR of all subjects and all feedback conditions).

#### Temporal metrics showed benefit of hybrid but not of continuous feedback

The load and release phase duration, as well as the trial duration, exhibited a similar trend to that seen for the delay between grip and load forces: *Discrete* and *Hybrid* feedback allowed for faster executions in subjects S1 and S2 compared to *no-feedback*, although this trend was not always significant (Fig. 4 and Movie S1). On average, *Hybrid* compared to *no-feedback* resulted in a 30% reduction of the median load phase, from 460:450 ms to 320:170 ms, in a 5% reduction of the median release phase, from 580:485 ms to 550:450 ms, and in a 16% reduction of the median trial duration, from 2.61:0.91 s to 2.19:0.49 s. *Discrete* feedback compared to *no-feedback* resulted in a 17% longer median load phase (540:675 ms), in a 17% shorter median release phase (480:365 ms), and in a 8% shorter trial duration (2.39:1.08 s). *Continuous* feedback consistently increased these temporal metrics to 665:660 ms (+45%), 800:485 ms (+38%), and 3.23:1.38 s (+24%) for median load phase, release phase and trial duration, respectively. Individually, these findings were all significant for S1, but for S2 the difference was only significant for *Discrete* feedback. Nonetheless, the results with *Hybrid* feedback were still lower for all three metrics for S2. As before, S3 did not profit from feedback, but, for this subject, *Hybrid* feedback was the only one not significantly increasing trial duration compared to *no-feedback*.

#### Summary of experiment 1

The PLT results under certainty showed that the *Hybrid* and *Discrete* feedback generally improved the temporal metrics for S1 and S2. *Hybrid* feedback also allowed for a more linear relation between grip and load forces for S1 and S2, who benefited most from sensory feedback. S3 showed highly coordinated control without feedback and did not improve in any of the considered metrics when provided with feedback. Instead, feedback worsened coordination for S3 in most cases, but *Hybrid* seemed to interfere the least. Notably, both S2 and S3 were slowed down by *Continuous* feedback, and S1 was significantly faster using any kind of feedback.

### Experiment 2: motor coordination under uncertainty

In order to examine the effect of sensory feedback under uncertainty, subjects performed a second experiment with the PLT, using the same non-breakable object, for three sessions of 20 repetitions each. However, in this experiment the weight of the object could change randomly and unexpectedly between 200 g, 300 g, and 400 g, from one repetition to the next. Subjects were informed that after a weight change, the object would stay the same weight for at least two further repetitions.

**Figure 5:**
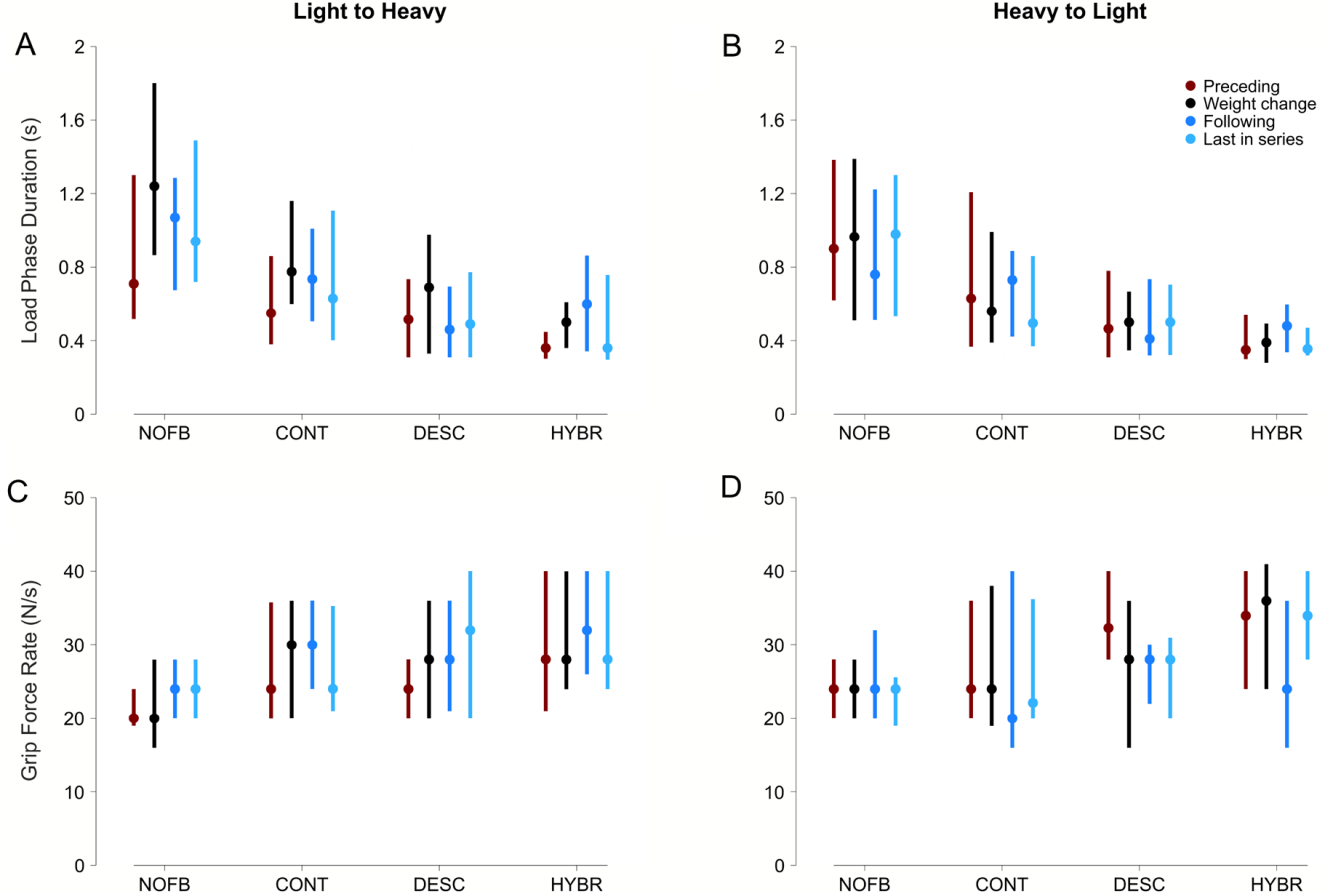
Experiment 2, results from the pick and lift test with unexpected weight changes. All graphs show boxplots (median and interquartile range) of the pooled data from all subjects. For each of the sensory feedback mode, the boxplots represent the trials preceding the weight change (red), the trials where the weight was changed (black), the trials immediately following the weight change (blue), and the last trials of each series of consecutive equal weights (light blue). Load phase duration for weight changes from lighter to heavier **(A)** and from heavier to lighter **(B)**. Maximum grip force rate, as the peak value of the differences between each grip force measurement during the load phase for weight changes from lighter to heavier **(C)** and from heavier to lighter **(D)**.

We analyzed variations on grasping behavior owing to unexpected weight changes by contrasting (1) the trial preceding the weight change (old weight), (2) the trial in which the weight changed (new weight), (3) the trial immediately following the weight change, and (4) the last trial in the series of consecutive lifts with the new weight. Clearly, each trial preceding a weight change is at the same time the last of the previous series of lifts, but the context may change. For the analysis, the weight changes were pooled for changes from lighter to heavier weights (from 200 g to 300 g or 400 g, and 300 g to 400 g), and for changes from heavier to lighter weights (from 400 g to 300 g or 200 g, and 300 g to 200 g). Considering previous findings by Jenmalm et al.^26^, our analysis focused on the load phase duration and the maximum grip force rate (Fig. 5). The subjects’ individual results are included in the Supplementary Materials as Figures S2, S3, and S4.

#### Feedback increased speed of lifting under uncertainty

It has previously been observed in non-amputees that a sudden increase in weight significantly increases the duration of the load phase in a PLT^20,26^. Indeed, we observed that the load phases became longer in the weight change trials for all feedback modes and then decreased again towards the last lift in the series as the same weight was lifted repeatedly (Fig. 5A; see also Figure S6—Supplementary Materials). This trend was statistically significant only for S1 using *Discrete* feedback (p=0.02 for preceding vs weight change; Figure S2A—Supplementary Materials). When considering the pooled data in *no-feedback* mode for changes from lighter to heavier weights, the load phase duration increased from 710:783 ms to 1240:938 ms (+75%), and then decreased to 940:770 ms (− 24% compared to weight change) in the last trial in the series with the same weight. Subjects exhibited generally shorter load phases when provided with tactile feedback, with the shortest observed using *Hybrid* feedback. When the weight was unexpectedly increased, the load phase duration with *Hybrid* increased from 360:145 ms to 500:250 ms (+39%) and increased again to 600:520 ms (+20% compared to weight change) in the following trial. Towards the last trial in the series, it decreased to 360:460 ms (− 28% compared to weight change).

#### Sudden weight decreases had no strong effect on maximum grip force rate

Previous work predicted a significant decrease in the grip force rate during the load phase between trials with an unexpected decrease of weight, and those following the weight change^26^. Even though this trend was confirmed with *Continuous* and *Hybrid* feedback, it was statistically significant only for S3 with *Hybrid* (p<0.001 for weight change vs following; Figure S4D—Supplementary Materials). In general, when using *Discrete* feedback, the grip force rate already decreased during the weight change trial and stayed low until the last trial in the series (Fig. 5). We also found a general trend for higher grip force rates with *Hybrid* and *Discrete* than with *no-feedback* (e.g., *Hybrid* 34.0:16.0 N/s and *Discrete* 32.3:12.0 N/s compared to *no-feedback* 24.0:8.0 N/s, for all trials preceding a sudden weight decrease). This complements the longer load phase durations observed with *no-feedback*.

As in Experiment 1, the maximum grip forces applied to the object were contained with all feedback modes (Figure S5—Supplementary Materials). As expected, the grip force rescaled according to the object weight similarly for all modes, except for *Discrete*.

#### Summary of Experiment 2

The PLT results from Experiment 2 showed that our subjects were faster when provided with any sensory feedback mode, but particularly with *Hybrid* mode. We found shorter load phase durations and higher grip force rates with tactile feedback than with *no-feedback* (Fig. 5 and Movie S1). Moreover, the subjects generally used a slower control approach under uncertainty, more feedback-based than what observed in Experiment 1. On average, the load phase, release phase and trial durations were 34% (496:488 vs 664:775), 16% (603:446 vs 697:573) and 10% (2.60:0.96 vs 2.86:1.45) longer, respectively.

### Qualitative evaluation of sensory perception

Subjects assigned qualitative descriptors to the elicited sensations using a questionnaire in which they could choose up to 16 descriptors for each stimulation mode (Table 2). The *Continuous* mode was described by all three subjects as an “electrical” sensation. This mode was also associated with the terms “pressure” (S1, S2), “buzzing” and “vibration” (S1, S3), “tingling” (S2), as well as “touch”, “needle prick”, “numbness” and “movement” (S1). The *Discrete* mode was described as “electrical” (S1, S2) and “pressure” (S2, S3), whereas terms like “tingling” (S2), “touch” and “tapping” (S3), and “needle prick” (S1) were each used once. The *Hybrid* mode was described as “electrical” and “buzzing” by all three subjects. Two times “pressure” (S2, S3) and “movement” (S1, S2) were used to further describe it, whereas “tingling” (S2), “vibration” (S3), “needle prick”, “tickling” and “itch” (S1) where each used once. Regarding the perceived intensity, interestingly the *Continuous* mode was rated as the most intense by all three subjects. *Hybrid* was rated equally as intense as *Discrete* by two out of three subjects and more intense than *Discrete* by one subject.

**Table 2:**
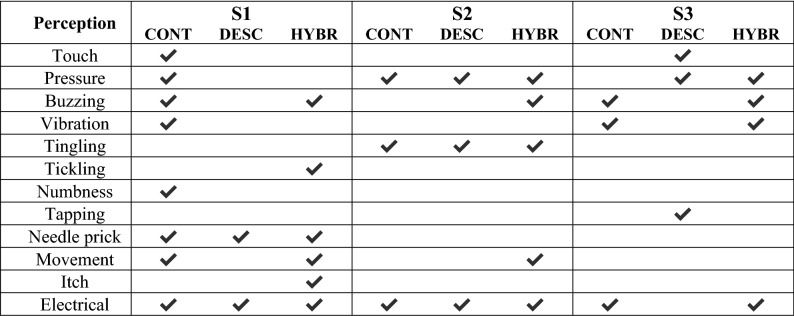
Subjective qualitative description of the perception for each stimulation mode.

**Figure 6:**
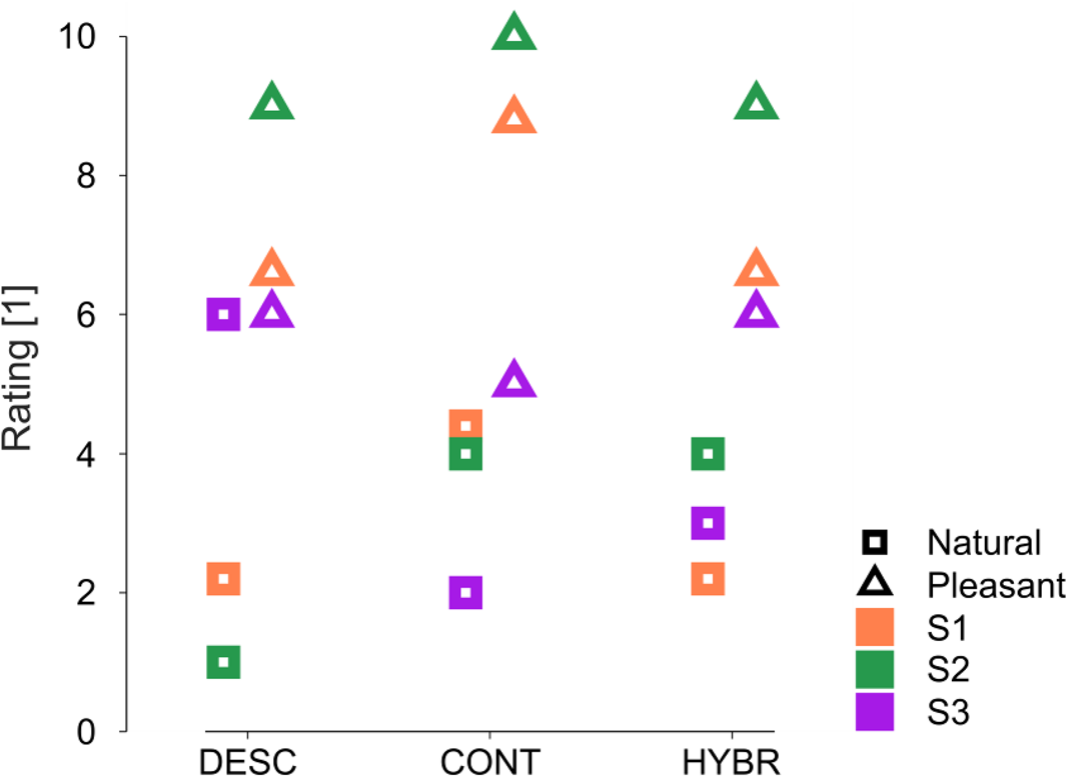
Subjective naturalness and pleasantness ratings. Squares represent the naturalness and triangles the pleasantness ratings for each of the three feedback modes. Each subject rated each sensory feedback mode once. Higher values correspond to more natural/pleasant ratings.

Moreover, subjects rated the naturalness and the pleasantness of each sensory feedback mode on a freely chosen, continuous scale (Fig. 6). The subsequently normalized results of the subjective naturalness rating (on a scale of 0 to 10, with 10 being perfectly natural) were: 3.5±1.3 (mean ± standard deviation) for *Continuous*, 3.1±2.6 for *Discrete*, and 3.1±0.9 for *Hybrid* feedback. Regarding the pleasantness of the stimulation, the subjective ratings were (on a scale from 0 to 10, with 10 being extremely pleasant): 7.9±2.6 for *Continuous*, 7.2±1.6 for *Discrete*, and 7.2±1.6 for *Hybrid*.

## Discussion

Feed-forward control of a myoelectric prosthesis relies on the user’s internal representation, or internal model^20,31^, of how to perform tasks such as reaching and grasping. In unimpaired subjects, the internal model is constantly updated using somatosensory information provided by intact biological sensors. Users of myoelectric prostheses deprived of somatosensory feedback cannot properly update such internal models, resulting in a poorer control experience^32,33^. Existing literature in this field suggests that open-loop control might be sufficient to retain internal models^34–38^. However, in previous work we found that, despite being provided with a high-resolution control interface (via implanted electrodes) and incidental sensory feedback richer than in conventional socket prostheses (osseoperceptive in addition to visual and auditory)^39^, prosthetic users did not retain a natural linear relationship between grip and load forces during an object lifting task. Here, we found that, by supplementing incidental sensory feedback with artificial tactile feedback via direct neural stimulation, a closer-to-normal grasping behavior was observed. The reason for this improvement could be the immediate benefit of tactile information provided in real-time, but also that the internal model was effectively updated by the feedback in the initial familiarization stage. The task used in this experiment was a non-variant repetitive exercise commonly performed with pure feed-forward control soon after a short familiarization stage. According to the Bayesian models of sensorimotor learning, such a fast adaptation might indicate lower uncertainty in the sensory feedback available in the moment (i.e., the compound sensory information from all channels available along the task) than in the state estimate or forward motor prediction^23–25^. The performance of S3 supports this hypothesis since he was already a skilled prosthetic user with potentially strong feed-forward control for whom tactile feedback had no positive effect. However, our results are limited by the number of subjects and further experiments on this regard are required to evaluate the effect of different neurostimulation modalities on updating internal models. In addition, the object was grasped through activation of the prosthetic hand but lifted without the assistance of the elbow joint. A person with an intact biological arm would employ a synergic activation of elbow and shoulder joints, whereas our participants could only rely on shoulder movements. Using exclusively shoulder movements for lifting an object is the usual way to conduct such tasks in long-term transhumeral prosthetic users, thus becoming their new norm. The behavior of our participants as seen in Fig. 3 seems to confirm this: the grasp and lift coordination of S1 and S2 in the *no-feedback* condition is as expected for prosthesis users, and the *Hybrid* feedback affected this grasp-lift coordination. S3, on the other hand, displayed skilled grasping coordination also without feedback. However, further work in transradial amputations should be conducted to confirm our findings.

The findings from our first experiment agree with and complement the results from a recently published case study by Clemente *et al.*, who provided a single subject with intraneural sensory feedback while performing the PLT^40^. This subject showed a reduced median delay between grip and load forces from 320 ms to 211 ms, when transitioning from *no-feedback* to neural feedback using the equivalent of our *Continuous* stimulation approach. Our subjects showed a considerably shorter median delay of 226 ms with *no-feedback*, which was further reduced to 176 ms using the *Hybrid* stimulation approach. Regarding the load phase duration without sensory feedback, our subjects showed a median of 460 ms in comparison to 480 ms in the single case study 40. This metric was reduced to 320 ms in our study and 330 ms in the single case study, both approaching near natural levels of 300 ms in non-amputee subjects^20^. The better performance of our subjects in the *no-feedback* condition, despite their disadvantageous level of amputation (transhumeral versus transradial), can be attributed to the difference in control interface. Whereas the subject in the single case study used surface electrodes for control and socket suspension for mechanical attachment, our subjects had a more reliable and higher-resolution control interface using implanted electrodes and direct skeletal attachment, which has been shown to improve controllability of prosthetic hands^3,18^. However, the two studies were characterized by several differences (e.g., type of implanted electrodes, level of amputation, prosthesis used, location of referred sensations) and thus we call for additional comparisons between the two control interfaces to confirm our hypothesis.

Our second experiment is comparable to the work of Jenmalm et al*.*^26^ with non-amputees, and it was designed to quantify the subjects’ reaction to unexpected changes of the object’s weight and the resulting feed-forward control uncertainty. We found that tactile feedback allowed faster control than *no-feedback* (shorter load phases and higher grip force rates), which can be interpreted as an indication that such feedback facilitated compensatory reactions after unexpected weight changes. When changing from a lighter to a heavier object, a significant increase in the duration of the load phase was expected^26^. Whereas we could observe this trend in all feedback modes, it was only statistically significant for S1 using the *Discrete* stimulation mode (p=0.02). This was likely due to the much smaller difference between the weights used in our experiment. Jenmalm et al. used weights of 230 g and 830 g, a more than threefold increase. Instead, in our case, the weights were either 1.5 or 2 times heavier, and thus a small increase in grasping force already sufficed to lift the heavier object. Furthermore, the subjects performed this part of the experiment with a more feedback-driven approach than in the first experiment, carefully processing the information related to the weight changes and the completion of the task. This argument is supported by the observation that all subjects were slower in all temporal metrics in the second experiment. Specifically, load phases were considerably longer than those expected with confident feed-forward grasping (see analysis of Experiment 1). Feedback-based grasping also decreased the “surprise” effect of the unexpected weight change (see also Figure S6—Supplementary Materials). Regarding the maximum rate of change of the grasping force during the load phase, Jenmalm et al. did not observed significant differences between trials with sudden weight increases, and neither did we. Interestingly, the grip force rates were generally higher with tactile feedback (especially with *Discrete* and *Hybrid*) than with *no-feedback*, suggesting that feedback made the subjects more confident during grasping. This is the first time that neural feedback has been found to improve grasping performance under uncertainty in a systematic and well-known assessment highly representative of daily prosthesis functionality, and importantly, with direct reference to results from similar test performed with non-amputee subjects. From a clinical point of view, this is of pivotal importance because unpredicted situations arise continuously during activities of daily living. Moreover, as particularly underlined by S3, the amputees may already be experienced enough not to require additional feedback to perform a task in predictive conditions, and thus the effectiveness of feedback interfaces may be reduced in these scenarios. Curiously, S3 seemed to perform even better than S2, although both are skilled prosthesis users. This may, in part, be due to S3 having more intuitive control via TMR; yet, S1 also received TMR and did not show comparable performance to S3. Bayesian models would suggest that S3’s confidence in his motor estimate is higher than his confidence in the information received via the sensory feedback (within the limited time-frame of this study). This stresses the importance of testing new systems with experimental setups resembling real-life, functionally relevant tasks as much as possible, and moving away from paradigms that represent uncommon prosthetic usage (e.g., manipulation and discrimination tasks in the absence of visual feedback).

One could argue that the *Hybrid* mode outperformed the *Continuous* mode because, in the latter, the amplitude modulation started incrementally from the perception thresholds. Beginning the provision of feedback at perception threshold implicates that the first contact was felt at the weakest, just noticeable intensity. A caveat with this approach is that stimulation, which is clearly perceived during investigation of the perception threshold when the subject is seated and focused, may not be perceivable during active object manipulation. The cognitive effort required by performing pick and lift repetitions may have hindered perception at the initial stage of interaction with the object, where the amplitude of the stimulation was proportional to the grasping force, and therefore low. The fact that using *Continuous* stimulation feedback slowed down subjects S2 and S3 may be evidence for such effect, arguably because subjects waited for the stimulation to become more clearly perceivable before proceeding with the lifting phase. In contrast, *Hybrid* mode, with the bio-inspired initial burst of pulses delivered at contact^20^, avoided this problem and possibly guided the subjects’ attention to the subsequent continuous feedback. The *Discrete* mode, even if seemingly better than *Continuous*, did not outperform *Hybrid*, which suggests that inferring knowledge about grip force continuously might be beneficial. The *Hybrid* mode we employed roughly resembled more sophisticated biomimetic models that were published recently and developed independently^41^. In two recent case studies of one^42^ and two^43^ subjects, neurostimulation with said models that consider aggregated nerve activity was perceived as more “natural”^43^ and was found to improve dexterity^42,43^ over amplitude modulation strategies comparable to the *Continuous* mode employed here. However, given the low number of subjects examined, additional studies are needed in order to generalize any finding to a wider range of functional tasks.

In agreement with previous studies on sensory feedback via direct nerve stimulation^3,44–46^, the qualitative experience of the perceived sensations was described as paresthesia, invoking “electrical”, “tingling”, or “buzzing” qualities. Similar qualities have been observed in transcutaneous electrical stimulation^27,47^. Whereas certain modes of pulse modulation have been described to transform paresthesia to more natural qualia^6^, these findings have not been replicated yet^48^. However, in addition to the aforementioned paresthetic sensations, our subjects also reported more natural sensations such as “pressure” and “touch”, which agrees with reports by other groups^42,44,45,49,50^. The presence of sensations recognizable as natural might be the cause for subjects reporting a “possibly” or “almost” natural experience^51^. Another reason might be the fact that although the quality of the sensations is masked by concurrent feelings of paresthesia, it is nevertheless somatotopically appropriate, and therefore intuitive and natural in this regard. This is of paramount importance as it suggests that providing intuitive sensory feedback via neural stimulation that is perceived as natural in location although not in quality, still provides considerable functional advantages in the control of limb prostheses^52^.

Interestingly, although the naturalness of the supplied sensory modes was rather low, the pleasantness was rated highly. In fact, after receiving the *Continuous* mode, S1 stated: “I want the feedback. […] This feeling would be nice for grasping vegetables or touching people. […] [It] was especially useful while lifting the heavy objects”. Moreover, S2 referring to the breakable objects during the familiarization stage for *Hybrid* mode stated: “I know just before the heavy object is going to break, because I can feel that I am grasping too hard”. There may be a small trend linking pleasantness and usefulness of the feedback. S1 and S2, who seemed to profit from the feedback, rated the pleasantness higher (S1 mean: 7.3, S2 mean: 9.3) than S3 (mean: 5.7) who did not seem to profit from the feedback. Overall, all subjects agreed on the idea that “[the feedback] does not need to be natural to be useful”. This mirrors previous findings showing that useful sensory feedback—even completely unnatural, modality mismatched vibrotactile feedback—is appreciated by prosthesis users^21,28^. There seems to be a strong demand for feedback, even if it is far from providing an experience as natural as an intact biological limb. In addition, since we aim to provide long-term feedback, and current nerve interfaces are limited in their ability to target specific afferents, it may be more important to focus on increasing pleasantness rather than naturalness, at least for now.

The limited number of subjects in this study did not allow for comprehensive statistical analyses. However, this is the first study that investigates the effects of invasive interfaces for the control of upper limb prostheses across three subjects using the same setup. Importantly, our results showed how the variability between only three subjects can already lead to different outcomes, and therefore we invite new studies that assess neuroprosthetics with larger cohorts of subjects than presently available.

## Materials and methods

### Subjects: e-OPRA and TMR

Three subjects with transhumeral amputation were recruited to participate in this study (referred to as S1, S2 and S3). More details about these subjects can be found in the work from Ortiz-Catalan et al.^53^, and Middleton and Ortiz-Catalan^54^. In brief, subjects S1 and S3 were implanted with the neuromusculoskeletal e-OPRA Implant System (Integrum, Sweden) in 2017, whereas S2 was implanted previously in 2013^3^. Additionally, S1 and S3 underwent a Targeted Muscle Reinnervation (TMR) surgical procedure aiming for intuitive myoelectric signals for hand opening and closing. Epimysial electrodes were implanted on biceps and triceps (naturally innervated and TMR) muscles and were accessed via the e-OPRA Implant System. Moreover, the e-OPRA included 4-contacts cuff electrodes located on the median nerve for S1 and S3 and the ulnar nerve for S2. The tests reported in this study were conducted around August 2018. All methods described here were carried out in accordance with the Declaration of Helsinki. The study was approved by the Swedish regional ethical committee in Gothenburg (Dnr: 769-12), and all subjects provided written informed consent.

### Prosthetic limb: sensors and control

The subjects performed the experiments while operating a custom prosthetic setup mechanically attached to the stump via a clamp mechanism over the percutaneous portion of the osseointegrated implant. The prosthetic setup comprised the IH2 Azzurra research robotic hand (Prensilia SRL, Italy), a standard prosthetic elbow (Ottobock, Germany), and the Artificial Limb Controller (ALC). The ALC is a custom-designed embedded system designed for closed-loop prosthetic control. It serves the dual purpose of recording EMG to control prosthesis movement and providing sensory feedback via neural stimulation^55^.

The IH2 Azzurra hand was controlled via direct control (also known as one-for-one control), consisting of the direct mapping of the speed of each movement to the mean absolute value of its corresponding channel (proportional speed control), calculated from 100 ms non-overlapping windows of EMG data sampled at 500 Hz. The EMG was high-pass filtered at 20 Hz, low-pass filtered at 250 Hz and notch-filtered at 50 Hz. The thresholds for direct control were customized for each subject to provide an optimal control of open and close movements of the hand in a tridigital grasp posture.

The hand was instrumented with six load cells aimed to track the tangential and the normal forces applied on thumb, index and middle fingers. The absolute values of the tangential and normal forces measured at the thumb, index and middle fingers were first summed to produce a unique positive combination value representative of the total force measured from the IH2 Azzurra hand. This value was compared against a contact-threshold primarily needed to mask inherent noise fluctuations of the six load cells. In the *Continuous* and *Hybrid* sensory modes, after overcoming the contact-threshold, the measured forces were then mapped to the nearest proportional stimulation amplitude value within the particular range of each subject. The processing delay between the instants of new information available from the fingertip sensors and the start of the stimulation was 19:27 ms (median:IQR).

### Experimental design

For all subjects, the experiment started with an extensive fitting session aimed to establish the subjects’ personal settings for both control and sensory feedback (further details are enclosed in the corresponding sections). The fitting session was then followed by a familiarization stage for each sensory feedback mode that consisted in grasping, lifting, moving, and repositioning breakable objects (blocks) from one side to the other of a 15 cm high wall. This had to be performed as fast as possible but without breaking the objects, similarly to the virtual eggs test^21^. The blocks were presented randomly and had different sizes, weights, and breaking forces (Figure S7—Supplementary Materials):

1. 200 g, 50 × 70 × 70 mm^3^, yellow, breakage at 4.90 N
2. 300 g, 50 × 70 × 85 mm^3^, red, breakage at 7.16 N
3. 400 g, 50 × 70 × 100 mm^3^, black, breakage at 9.38 N.

The familiarization stage was also used to verify the correct functioning of the ensemble of sensory feedback and control settings. The fitting session and familiarization stage lasted from 10 to 20 min per subject.

The PLT was used to assess the subjects’ motor coordination when using the prosthesis in the context of routine grasping (Experiment 1)^29^ and unpredictable grasping due to unknown object weights (Experiment 2)^30,56^. This test was conducted with a different test object, consisting of a 200 g 40 × 45 × 130 mm^3^ plastic block with three embedded load cells, two measuring the grip force applied perpendicularly to the grasping surfaces, and one measuring the load force applied tangentially on the object before lift-off (Fig. 1A). A magnetic latching mechanism commanded via a software interface allowed the silent connection of up to two extra weight appendices of 100 g each, resulting in the same weight combination as in the familiarization stage (200 g, 300 g, 400 g) (Figure S7—Supplementary Materials). No breaking thresholds were imposed during the two experiments. A single repetition of the PLT required the subject to (1) move the arm to reach the object, (2) grasp the object, (3) lift the object a few centimeters above the desk, (4) reposition the object on the table and finally, (5) release the object. Experiments 1 and 2 consisted of two and three sessions, respectively, with 20 repetitions each, and the subjects were instructed to perform the test at their own comfortable pace. In Experiment 1, the weight of the test object was always 200 g, while in Experiment 2 the weight of the test object was randomly changed in between lifts^26^. The second PLT experiment was intended to assess the motor coordination under uncertainty. The weight changes were masked and randomized, but the subjects knew that after every change, the weight would stay the same for at least two more repetitions. The maximum number of repetitions of a single weight was 5 (unknown to the subjects). The random order of weight changes was manually reviewed to ensure a balanced distribution between all weights and transitions. The second PLT experiment was intended to assess the motor coordination under higher uncertainty.

To assess the subjective qualities of the provided feedback modes, the subjects answered a brief questionnaire (Questionnaire S1—Supplementary Materials) once per feedback mode, after completing all the tests. The questionnaire started with a free description of the perception, to gather the impression of the perception without imposing any expectations on the subject. Then we asked if and how the perception could be reproduced on the contralateral limb to understand whether it could be compared to a natural one^57^. We aimed to qualify the perception via a list of 16 qualitative descriptors from which the subjects could choose as many as desired or even define new ones. This list was inspired by previous works^27,47^. Lastly, we asked the subjects to rate the subjective intensity, naturalness and pleasantness of the sensation on a freely chosen scale. For the analysis, the scores were converted to an 11-point (0 to 10) scale.

### Sensory feedback modes

The forces profiles measured from the sensors in the fingertips of the robotic hand were used to drive the neurostimulator in one of three different feedback modes (Fig. 1):

1. *Continuous* modulation (CONT) of the current amplitude, linearly proportional to the modulus of the grasp and load forces measured (see below). The pulses were delivered at a constant frequency of 30 Hz^58^, and the current amplitude was updated every 3 pulses (≈100 ms).
2. *Discrete* stimulation (DESC) with fixed parameters in correspondence with the events of touch and release^21^. The pulses were delivered at the constant current amplitude of 150% of the perception threshold and at the constant frequency of 100 Hz in burst of 5 pulses (50 ms).
3. *Hybrid* of the previous two modes (HYBR). Continuous modulation of the current amplitude as in mode 1 combined with burst of 5 pulses at higher frequency in correspondence with the discrete events of touch and release, as in mode 2.

These feedback modes were designed taking into considerations the current knowledge in sensorimotor control and the biologic involvement of Fast-Adapting (FA) and Slow-Adapting (SA) nerve fibers during the different phases of object manipulation in grasping-and-lifting tasks^20^. Furthermore, during *Continuous* and *Hybrid* mode, increasing pressure on the fingertips resulted in increased stimulation, meaning increased recruitment of nerve fibers, and led to an increased area of stimulation, as it would with natural hands^59^. We therefore present these sensory feedback modes as biologically inspired, and it is not within the scope of this study to assess whether or not these modes resembled the biological firing of FA and SA nerve fibers. The scope of the study is intended to be about the functionality of prosthetic control, and in particular about the motor coordination as the relation between the grip and the load forces applied during grasping-and-lifting interactions with an object.

For the sake of comparison, the PLT was performed with each sensory feedback mode as well as in the *no-feedback* mode. The order of execution of the different modes was randomized for each subject (Table S1—Supplementary Materials), and we tried to leave at least 4 h between each mode, whenever this was possible. The subjects were blind to the sensory feedback mode in use and their order of execution, and the logic behind the modes was not explicitly explained to them.

### Sensory characterization and neural stimulation

The neural stimulation was based on cathodic-first, rectangular, bipolar (50 µs inter-pulse delay), asymmetric (10:1), charge-balanced, current-controlled, electric pulses. A standardized summary of stimulation parameters as suggested by Günter et al*.*^58^ is available in Table S2 of Supplementary Materials. Only one contact of the cuff electrode per subject was used for stimulation. This contact was found after a complete characterization of all contacts in terms of impedance, minimum and maximum perception thresholds, and location of somatotopic perception. The impedance was measured with an embedded automatized routine that stimulated the contact with sinusoidal currents (at 500 and 1000 Hz) and sampled the resulting voltages. These results were then visually confirmed with a similar manual procedure done with an oscilloscope. The minimum perception threshold was found via subjects’ direct feedback after single pulse stimulations. The subjects were comfortably sat in front of a computer and asked to report whether they perceived a given neural stimulus or not. The pulses were delivered at different amplitude and width combinations, always moving from lower to higher charge.

For each subject, the pulse width used during the experiment was set to ensure perception of a single pulse. Once the pulse width was found, the maximum perception current amplitude threshold was selected by the subjects as the strongest stimulation that was still perceived as pleasant. The amplitude steps between minimum and maximum current limits were pre-set by the resolution of the stimulator, whose step-size depended on the current range. In particular, the step-size available was 10 µA for currents between 20 and 260 µA, 20 µA between 260 and 500 µA, and 50 µA between 500 and 1000 µA. Lastly, it was verified that all the amplitude steps were perceivable by the subjects. The verification consisted in delivering pulses from minimum to maximum current limits with increasing amplitude sequence and asking the subjects to count the perceived steps in between. This procedure was done twice per subject.

### Data analysis and statistical methods

For the first experiment with the PLT, the motor coordination was evaluated through the level of grip-load force coordination, quantified as the temporal delay between the instants when the grip and load forces reached 50% of the load force measured at lift-off. Moreover, we calculated other temporal metrics from the different phases of the PLT, namely the load phase duration, the release phase duration, and the trial duration (Fig. 1)^56^. For the second experiment with the PLT, in which the weight of the test object was randomly changed between trials, the focus was moved to the effect of sensory feedback under two types of uncertainties: sudden weight increases, and sudden weight decreases. The metrics and the analysis used for this part of the study were inspired by similar experiments with biological hands by Jenmalm et al.^26^. Specifically, we analyzed the weight changes dividing them into "lighter to heavier" and "heavier to lighter" groups. Corrective actions (i.e., specific behavior) triggered by an unexpected weight change in a certain “direction”, were the same irrespective of the weight itself. Then, we compared the trials preceding the weight change, the trials where the weight changed, the trials following the weight change, and the last trials in each series of consecutive same weights. The metrics were the load phase duration and the maximum grip force rate, calculated as the peak value of the differences between each grip force measurement during the load phase.

All datasets were analyzed using the built-in statistics functions of MATLAB 2018a (MathWorks, USA). Considering the limited number of subjects, the statistical tests were performed only from data within the same subject. However, the pooled data from all subjects were shown to simplify the visualization, especially for the second experiment. The one-sample Kolmogorov-Smirnov test (p > 0.7) was used to verify the normality of the distributions of the datasets. Since the datasets exhibited both normal and non-normal distributions, ANOVA or Kruskal-Wallis tests were used, depending on the case, followed by a multiple comparison test with Bonferroni correction. For all cases, a p value lower than 0.05 was considered as reference for statistical significance. In the figures, the statistical significance is reported according to the following notation: * = p < 0.05, ** = p < 0.01, *** = p < 0.001.

## Supplementary information

Supplementary file1 (PDF 799 kb)

Supplementary file2 (MP4 84336 kb)

## Data Availability

Data and materials produced during this study can be made available upon reasonable request.
