# Supplementary material for: Neural feedback strategies to improve grasping coordination in neuromusculoskeletal prostheses
Source: Sci Rep. 2020 Jul 16;10:11793. doi: 10.1038/s41598-020-67985-5 (PMC7367346; doi:10.1038/s41598-020-67985-5)
Supplement: Supplementary file 1 — Supplementary file1 (PDF 799 kb) [file 41598_2020_67985_MOESM1_ESM.pdf]

Title

Neural feedback strategies to improve grasping coordination in neuromusculoskeletal prostheses

Authors

Enzo Mastinu*, Leonard F. Engels*, Francesco Clemente, Mariama Dione, Paolo Sassu, Oskar Aszmann, Rickard Brånemark, Bo Håkansson, Marco Controzzi, Johan Wessberg, Christian Cipriani, and Max Ortiz-Catalan

SUPPLEMENTARY MATERIALS

Movie S1. Neural feedback strategies to improve grasping coordination in neuromusculoskeletal prostheses.

Results:
Fig. S1. Experiment 1, additional results from the pick and lift test with same weight.
Fig. S2. Experiment 2, subject1: individual results from the pick and lift test with unexpected weight changes.
Fig. S3. Experiment 2, subject 2: individual results from the pick and lift test with unexpected weight changes.
Fig. S4. Experiment 2, subject 3: individual results from the pick and lift test with unexpected weight changes.
Fig. S5. Experiment 2, maximum grip force of all trials with equal weights.
Fig. S6. Experiment 2, grip force from contact to lift-off for trials preceding a weight change and those immediately following.

Materials and Methods:
Fig. S7. Experiments equipment.
Questionnaire S1. Questionnaire for subjective naturalness and pleasantness ratings.
Table S1. Randomized order of execution for sensory feedback modes.
Table S2. Stimulation parameters.

SUPPLEMENTARY FIGURES

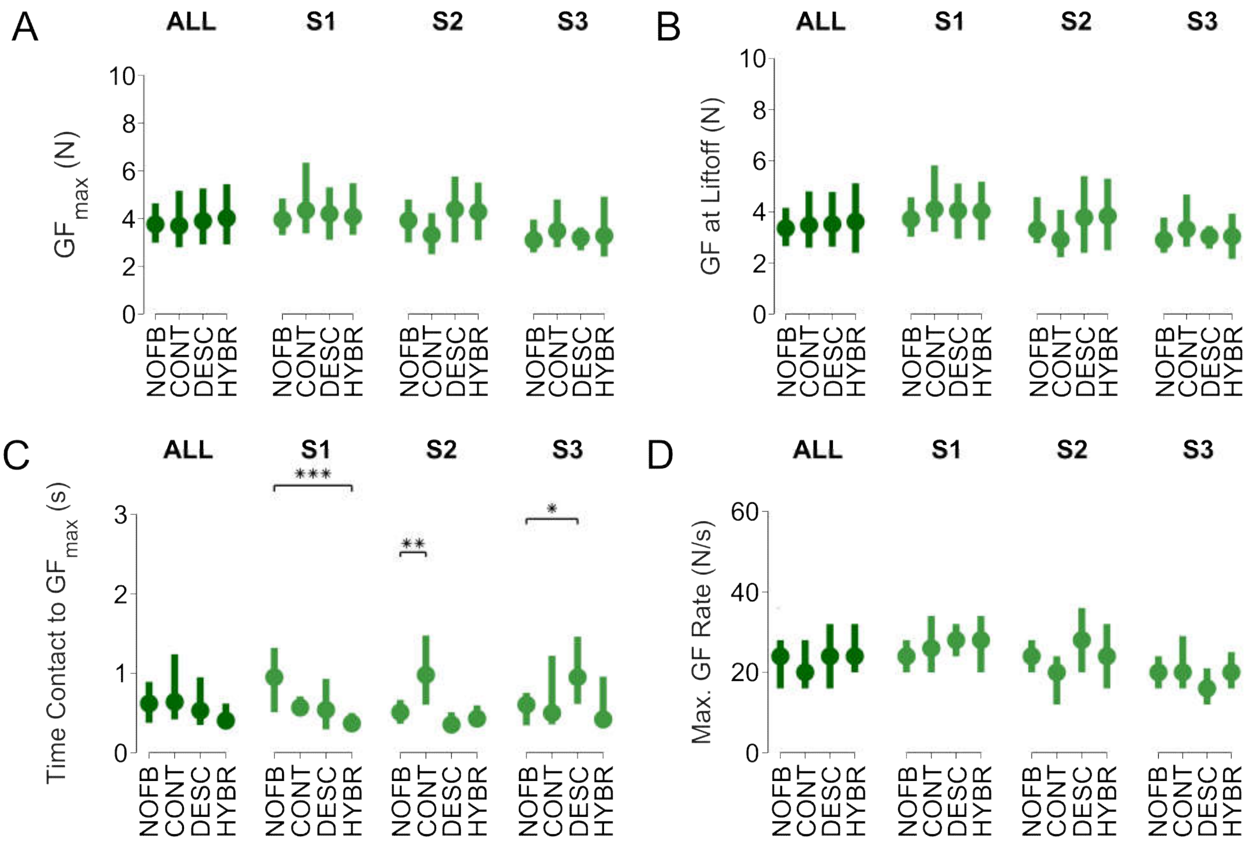

Figure S1. Experiment 1, additional results from the pick and lift test with same weight. All graphs show boxplots (median and interquartile range) of the pooled data on the left side and the individual results on the right side for all sensory feedback modes. The statistical significance is reported according to the following notation: * = $p<0.05$, ** = $p<0.01$, *** = $p<0.001$. A) Maximum grip force as the peak value of the grip force applied to the object during execution. B) Grip force at the instant of lift-off of the object. C) Time to reach the maximum grip force from the instant of the first contact with the object. D) Maximum grip force rate, calculated as the peak value of the differences between each grip force measurement during the load phase.

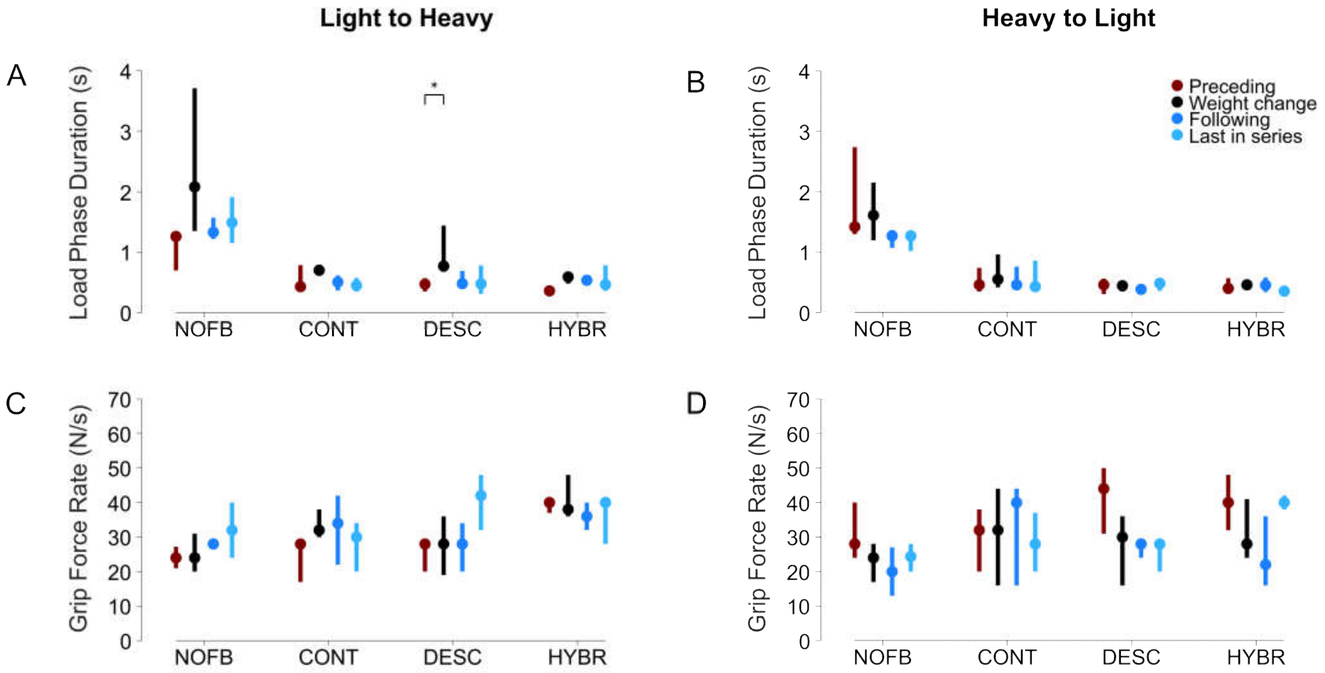

Figure S2. Experiment 2, subject 1: individual results from the pick and lift test with unexpected weight changes. For each of the sensory feedback modes, the boxplots represent the trials preceding the weight change (red), the trials where the weight was changed (black), the trials immediately following the weight change (blue), and the last trials of each series of consecutive equal weights (light blue). Load phase duration for weight changes from lighter to heavier (A) and from heavier to lighter (B). Maximum grip force rate, as the peak value of the differences between each grip force measurement during the load phase for weight changes from lighter to heavier (C) and from heavier to lighter (D).

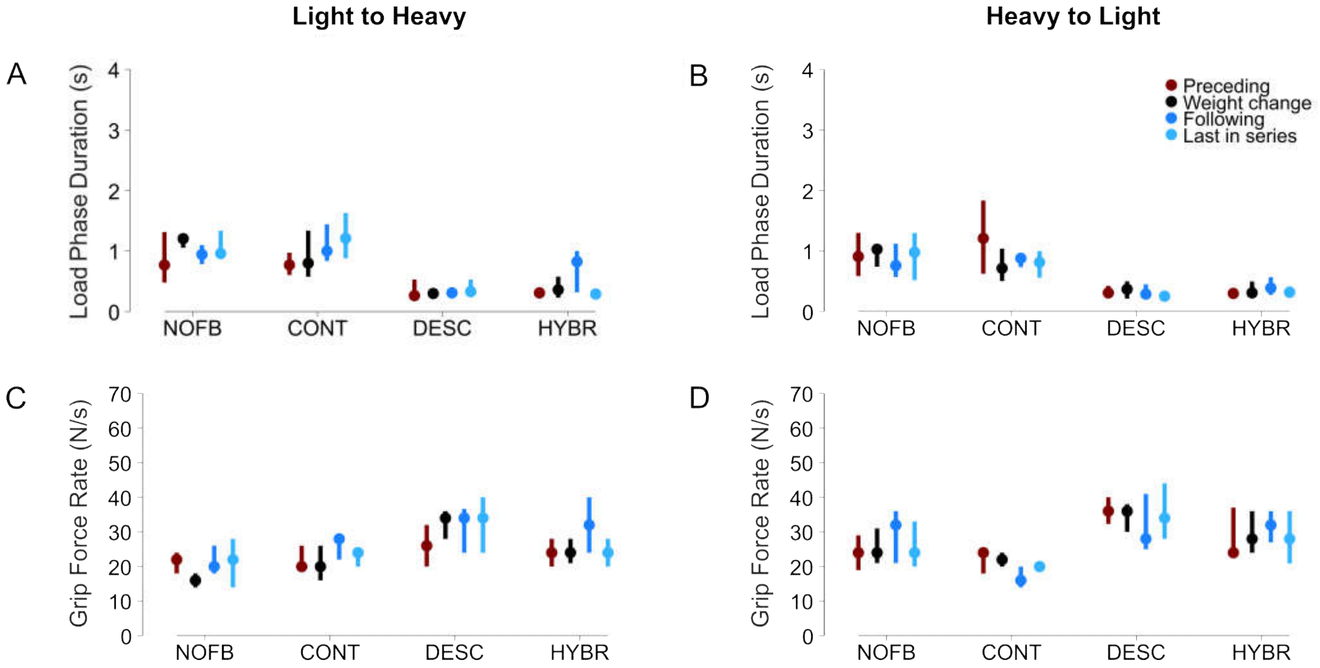

Figure S3. Experiment 2, subject 2: individual results from the pick and lift test with unexpected weight changes. For each of the sensory feedback modes, the boxplots represent the trials preceding the weight change (red), the trials where the weight was changed (black), the trials immediately following the weight change (blue), and the last trials of each series of consecutive equal weights (light blue). Load phase duration for weight changes from lighter to heavier (A) and from heavier to lighter (B). Maximum grip force rate, as the peak value of the differences between each grip force measurement during the load phase for weight changes from lighter to heavier (C) and from heavier to lighter (D).

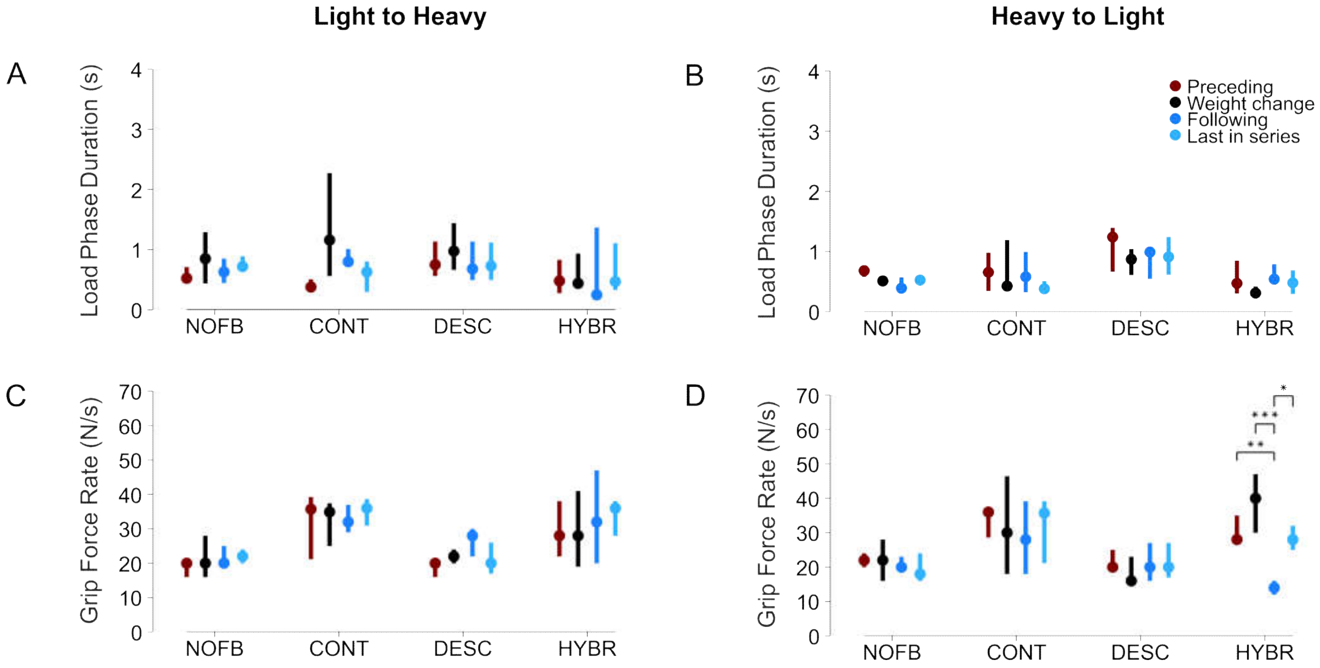

Figure S4. Experiment 2, subject 3: individual results from the pick and lift test with unexpected weight changes. For each of the sensory feedback modes, the boxplots represent the trials preceding the weight change (red), the trials where the weight was changed (black), the trials immediately following the weight change (blue), and the last trials of each series of consecutive equal weights (light blue). Load phase duration for weight changes from lighter to heavier (A) and from heavier to lighter (B). Maximum grip force rate, as the peak value of the differences between each grip force measurement during the load phase for weight changes from lighter to heavier (C) and from heavier to lighter (D).

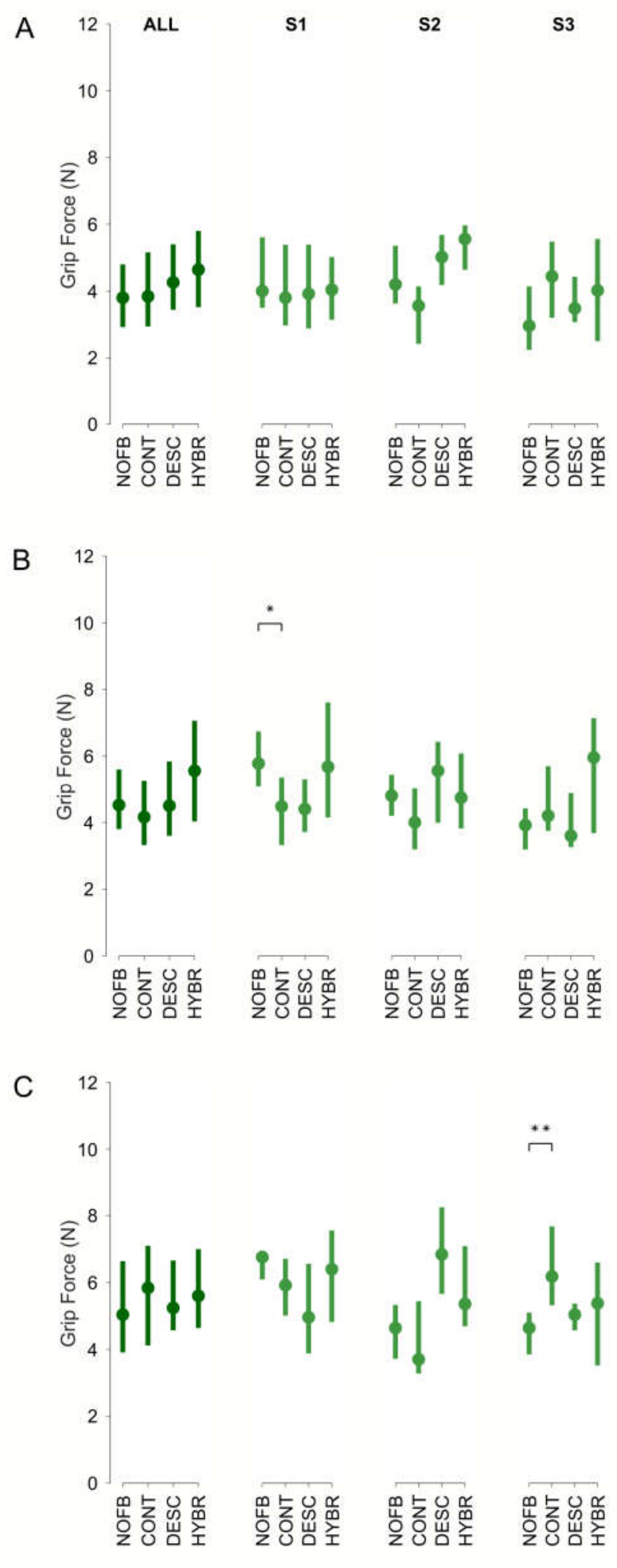

Figure S5. Experiment 2, maximum grip force of all trials with equal weights. For all weights, 200 g (A), 300 g (B), and 400 g (C), the left panel shows the pooled data of all subjects and the right panels show individual data for each subject. S1 applied significantly lower grip force to the 300 g object when provided with feedback than without (*Continuous*, $p<0.05$). S3 applied significantly higher grip force to the 400 g object when provided with feedback than without (*Continuous*, $p<0.01$).

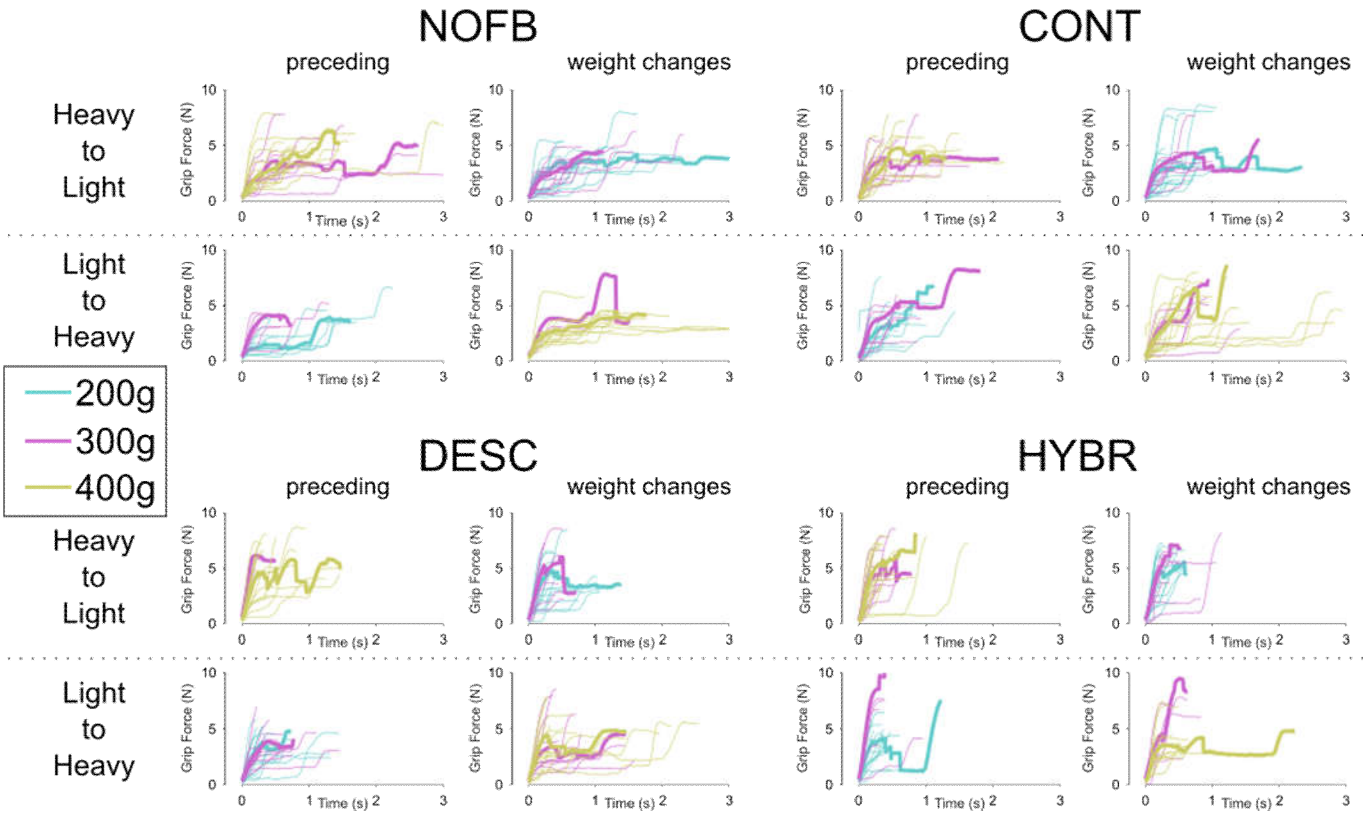

Figure S6. Experiment 2, grip force from contact to lift-off for trials preceding a weight change and those immediately following. Trials are separated by feedback mode and changes from heavy to light weights and light to heavy weights. All individual trials are shown as thin lines; bold lines depict the average grip force excluding outlier trials (as determined by Matlab's "rmoutlier" function). Colors represent object weights.

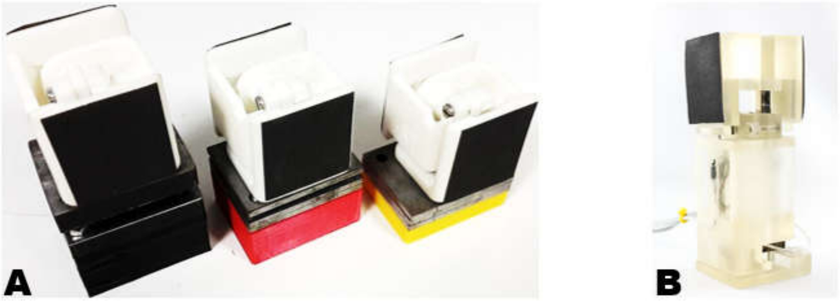

Figure S7. Experimental equipment. A) test-and-training objects with different combinations of weight, size and color. B) Instrumented test object used for the pick and lift test. A magnetic latching mechanism commanded via a software interface allowed the connection of up to two extra weight appendices of 100g each.

Table S1
Randomized order of execution for sensory feedback modes

ORDER of EXECUTION	S1	S2	S3
1	CONT day1	CONT day1	DESC day1
2	DESC day1	NOFB day1	NOFB day1
3	NOFB day1	HYBR day2	CONT day2
4	HYBR day1	DESC day2	HYBR day2

Table S2
Stimulation parameters

Label	Parameter	Value	Unit
A	Electrode surface area	0.8 to 1.6	mm^2
D	Duration of stimulating phase of a stimulus pulse	100 to 200	µs
D’	Duration of reversal phase of a stimulus pulse	10*D	µs
F	Stimulation pulse frequency	30 to 100	Hz
I	Current of stimulating phase	80 to 650	µA
I’	Current of reversal phase	I / 10	µA
N	Number of pulses per train	25 to 150	-
R	Train rate	Unknown	Hz
W	Inter-phase delay	50	µs
P	Total application time (per day)	< 7	hours
S	Effective stimulation time (per day)	< 1	hours
Y	Treatment duration	-	years

SUPPLEMENTARY METHODS

Questionnaire for Subjects

Subject ID	Date & Time

Sensory feedback mode	Pulse width	Amplitude	Frequency	Duration
Cuff Electrode info:				
Modulation Parameter:			Modulation Range:	

Notes:

1) Free description of the sensation.

2) How could you reproduce the sensation on your contralateral limb?

3) How would you describe the perceived sensation(s)? Check all that apply.

Touch	Pressure	Buzzing	Vibration	Tingling	Tickling	Pain
Numbness	Burning	Warm	Cold	Tapping	Needle prick	Movement
Itch	Electrical					

4) Where did you perceive the sensation(s)? Try to mark in the image below the perceived shape and size. Only for Continuous and Hybrid modes, mark the perceived shape and size with different colors for:
   - Minimum Parameter Modulation
   - Maximum Parameter Modulation

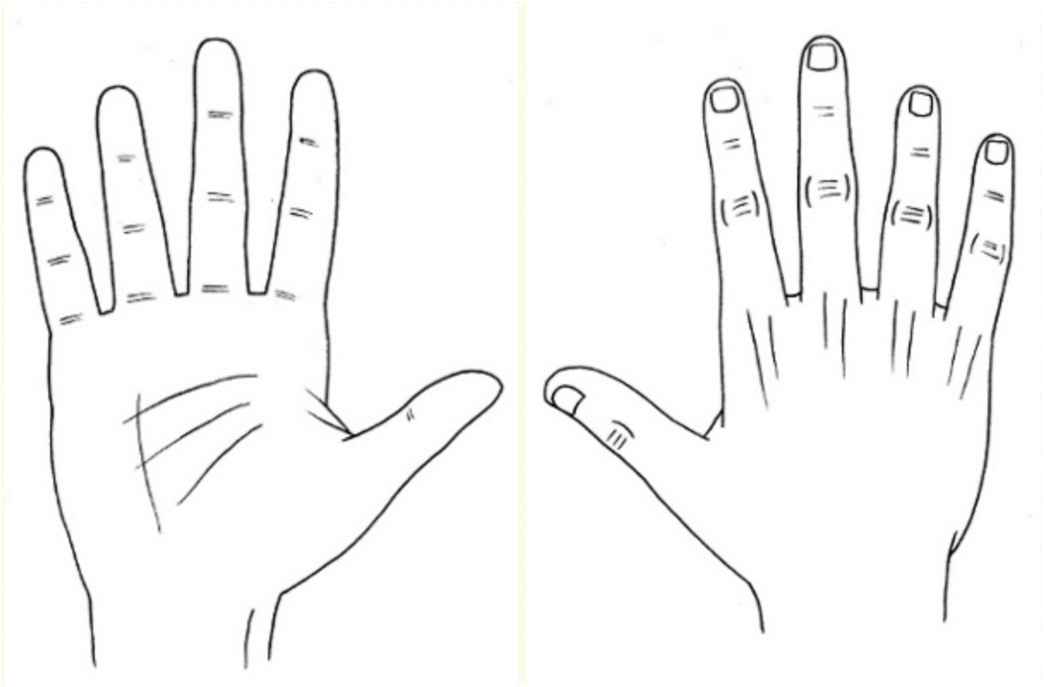

5) How intense/strong was the maximum stimulation perceived? *Define a scale for reference.*

Value	Relative Boundaries:	Min	Max

6) How (un-)natural was the sensation compared to actually being touched on your contralateral limb? *Define a scale for reference.*

Value	Relative Boundaries:	Min	Max

7) How (un-)pleasant was the sensation (imagine you would feel this sensation every time you grasp something)? *Define a scale for reference.*

Value	Relative Boundaries:	Min	Max
